# Epigenetic Aging of Critical Illness Survivors Assessed by the Muscle‐Specific “Clock” and Its Relationship With Reduced Long‐Term Muscle Strength

**DOI:** 10.1111/acel.70629

**Published:** 2026-07-12

**Authors:** Ceren Uzun Ayar, Inge Derese, Greet Van den Berghe, Ilse Vanhorebeek

**Affiliations:** ^1^ Laboratory of Intensive Care Medicine, Department of Cellular and Molecular Medicine KU Leuven Leuven Belgium; ^2^ Clinical Division of Intensive Care Medicine University Hospitals Leuven Leuven Belgium

**Keywords:** critical illness, DNA methylation, epigenetic aging, epigenetic clock, intensive care unit, muscle weakness, post‐intensive care syndrome, RNA expression

## Abstract

Critically ill patients requiring treatment in the intensive care unit (ICU) suffer from muscle weakness that persists for years. As compared with healthy subjects, skeletal muscle of patients biopsied five years post‐ICU revealed an abnormal transcriptome partially associated with poor muscle strength. We now hypothesized that skeletal muscle of long‐term ICU survivors is “epigenetically aged”, as determined by a muscle‐specific epigenetic clock, and that such accelerated epigenetic aging contributes to their long‐term muscle weakness. Muscle DNA‐methylation data from former ICU patients at 5‐year follow‐up (*N* = 118) and healthy controls (*N* = 160), aged 18–89 years, were analyzed by the MEATv2 epigenetic clock. First, epigenetic age (DNAmAge), epigenetic minus chronological age (AADiff) and epigenetic age acceleration (AAResid) were compared between 97 former patients and 97 controls, propensity score‐matched for age and sex. Next, the impact of any muscle‐specific epigenetic aging of ICU survivors was investigated, via multivariable models, as a potential contributor to the altered transcriptome and reduced muscle strength. Former ICU patients showed a significantly higher muscle DNAmAge, AADiff, and AAResid than matched controls. In adjusted models, higher muscle DNAmAge, AADiff, or AAResid did not substantially contribute to differentially expressed muscle RNAs in former patients as compared with controls and was not associated with the poor long‐term muscle strength. In conclusion, five years after ICU discharge, former patients showed accelerated epigenetic aging in skeletal muscle. However, the muscle‐specific epigenetic clock did not capture molecular changes that are associated with long‐term muscle weakness, which highlights the need for other muscle‐specific biological predictors of age‐related physical impairment.

**Trail Registration:** ClinicalTrials.gov: NCT00512122

## Graphical abstract

The MEATv2 muscle‐specific epigenetic clock captures biological aging, but not the molecular changes associated with long‐term muscle weakness in ICU survivors.

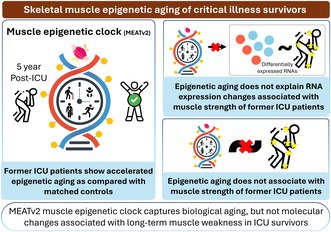

## 1 Introduction

Critically ill patients requiring intensive care are at high risk of developing multiple organ dysfunction. A significant proportion of these patients develop intensive care unit‐acquired weakness (ICUAW), a condition characterized by weakness of both limb and respiratory muscles (Vanhorebeek et al. 2020). ICUAW has been associated with increased mortality, a prolonged hospital stay and impaired functional recovery. Beyond the acute phase, many ICU survivors face persistent long‐term complications, collectively labeled post‐intensive care syndrome (Renner et al. 2023; Vanhorebeek and Van den Berghe 2023). Among these sequelae, persistent muscle wasting and weakness are especially prominent and substantially reduce quality of life (Hermans et al. 2019; Herridge et al. 2011). Contrary to the extensive mechanistic research into the pathophysiology of ICUAW, studies exploring potential molecular mechanisms underlying long‐term persistent muscle weakness are scarce (Friedrich et al. 2015; Vanhorebeek et al. 2020). In this regard, our earlier work revealed that even five years after critical illness, skeletal muscle from former ICU patients as compared with healthy controls displayed transcriptome alterations, of which many were associated with the patients' lower long‐term muscle strength (Uzun Ayar et al. 2025). The transcriptome alterations, especially those associated with reduced muscle strength and disrupted pathways such as mitochondrial function, lipid metabolism and fibrosis, were in part explained by long‐term alterations in DNA‐methylation (Uzun Ayar et al. 2026).

Both during and after an ICU stay, (former) critically ill patients are typically exposed to a wide range of events or factors that can affect the rate of biological aging, such as major trauma, physical stress, disturbed physical activity, systemic inflammation, metabolic dysregulation, and change in nutritional intake (Booth and Brunet 2016; Hahn et al. 2017; Jones et al. 2015; Kim et al. 2022; Rousseau et al. 2021; Seale et al. 2022; Sullivan et al. 2018). Altered DNA‐methylation is recognized as a primary hallmark of aging (Lopez‐Otin et al. 2023; Seale et al. 2022). DNA‐methylation patterns not only capture chronological age but also provide a molecular readout of biological aging by integrating the effects of environmental exposures (Feil and Fraga 2012). Epigenetic clocks, which estimate biological age by leveraging sets of age‐informative DNA‐methylation sites, are considered the most robust molecular estimators of aging (Horvath 2013; Jylhava et al. 2017). Several pan‐tissue clocks have been developed and validated, and deviation between epigenetic and chronological age has been linked to chronic disease, reduced lifespan, and impaired health span (Levine et al. 2018). Epigenetic aging, however, is tissue‐specific, with organs aging at variable rates (Rando and Wyss‐Coray 2021; Thompson et al. 2010). Therefore, a skeletal muscle‐specific clock (MEATv2) has been developed to predict chronological age from muscle DNA‐methylation data with high accuracy (Voisin et al. 2021).

**FIGURE 1:**
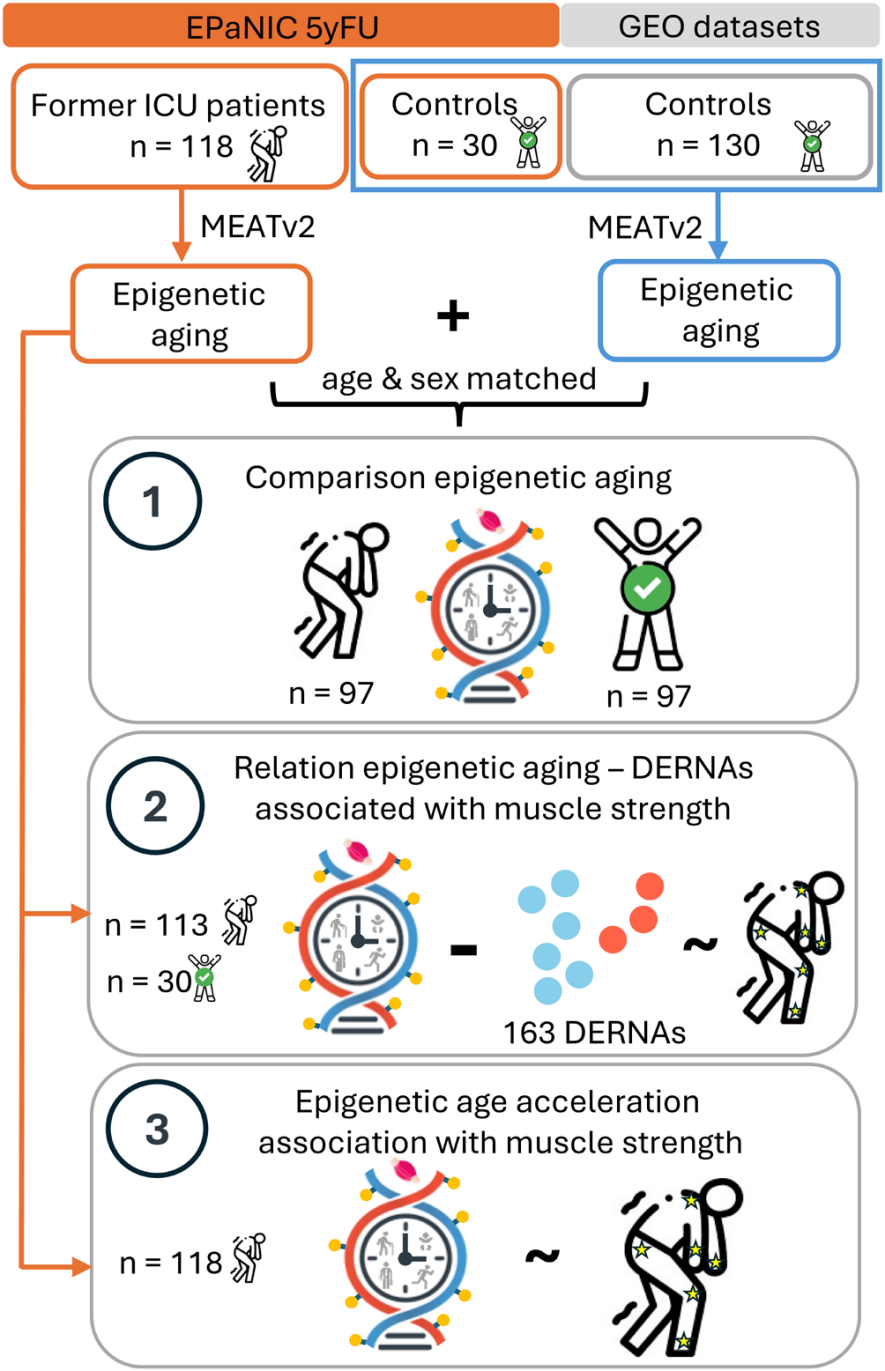
Study design. At the EPaNIC 5‐year follow‐up study, good quality skeletal muscle DNA‐methylation data had been obtained from 118 former ICU patients (Uzun Ayar et al. 2026). Good quality DNA‐methylation data obtained in the same study from 30 subjects who had never required an ICU admission were pooled with DNA‐methylation data of 130 healthy control subjects included in four publicly available datasets retrieved from the Gene Expression Omnibus (GEO) database, yielding a total pool of 160 control subjects. Measures of epigenetic age(ing) were calculated from these DNA‐methylation data with use of the MEATv2 clock. Step 1: Comparison of measures of epigenetic aging among former ICU patients and controls who were propensity score‐matched for age and sex (*n* = 97 per group). Step 2: Assessment of whether epigenetic aging may explain long‐term abnormal RNA expression associated with long‐term strength of the former ICU patients. We previously identified 163 RNAs that were differentially expressed in muscle of former EPaNIC patients 5 years after ICU admission (*n* = 115 of which 113 overlapping with the DNA‐methylation dataset) as compared with controls (*n* = 30) and associated with long‐term strength (hand, wrist, elbow, shoulder, hip, knee or ankle) of the former ICU patients (Uzun Ayar et al. 2025). Multivariable linear regression models were built for each of the 163 RNAs as dependent variable, with the epigenetic age(ing) marker added as independent variable in addition to age, sex, and BMI. Step 3: Association of epigenetic aging with muscle strength measures (hand, wrist, elbow, shoulder, hip, knee, or ankle) of the former ICU patients in multivariable models.

We hypothesized that years after critical illness, skeletal muscle of long‐term ICU survivors shows an epigenetic age acceleration when compared with matched controls, as determined by a muscle‐specific epigenetic clock, and that such age acceleration may in part explain the impact of critical illness on long‐term RNA expression in muscle and may associate with impaired long‐term muscle strength (Figure 1).

## 2 Methods

### 2.1 Study Participants

#### 2.1.1 Former Critically Ill Patients

This is a pre‐planned prospective secondary analysis of the multicenter EPaNIC trial (ClinicalTrials.gov‐NCT00512122) and its 5‐year follow‐up (Casaer et al. 2011; Hermans et al. 2019). This trial included 4640 adult critically ill patients to investigate the effect of early supplementation of insufficient enteral nutrition with parenteral nutrition as compared with omitting supplemental parenteral nutrition during the first week in ICU (Casaer et al. 2011). Long‐term physical morbidity of 674 EPaNIC patients not suffering from conditions that could confound the morbidity endpoints had been investigated 5 years after ICU admission (Hermans et al. 2019). From 120 of the former ICU patients, in vivo needle biopsies had been taken percutaneously from the musculus vastus lateralis of the quadriceps femoris at mid‐thigh level (Bergström technique, local anesthesia with lidocaine 2%). Good quality muscle DNA‐methylation data previously obtained from 118 of the former ICU patients, age range 24–80 years, were used for the present study (Figure 1) (Uzun Ayar et al. 2026).

#### 2.1.2 Control Subjects

Skeletal muscle (vastus lateralis) DNA‐methylation data from different cohorts of control subjects were pooled for this study. During the EPaNIC follow‐up study, we had recruited 50 individuals who had never required an ICU admission, with comparable age, sex, and BMI as the former ICU patients, of whom 31 donated a muscle biopsy. Good quality muscle DNA‐methylation data had been obtained from 30 of these control subjects (Figure 1) (Uzun Ayar et al. 2026). Combination with genome‐wide DNA‐methylation data of healthy subjects included in four publicly available datasets retrieved from the Gene Expression Omnibus (GEO) database (GSE114763 (Seaborne et al. 2018), GSE49908 (Day et al. 2013), GSE50498 (Zykovich et al. 2014), GSE38291 (Ribel‐Madsen et al. 2012)) yielded a total pool of 160 control subjects, with an age range of 18–89 years, for the current study. Information on the different datasets is given in Table S1.

#### 2.1.3 Propensity Score Matching of Former Critically Ill Patients and Control Subjects

As the total pool of 160 control subjects and the 118 former ICU patients differed in age and sex, propensity score matching for age and sex (1:1 nearest neighbor, caliper 0.2) was performed with the MatchIt package in R (v4.7.2) (Ho et al. 2011) for comparisons of former patients with controls.

### 2.2 Determination of Epigenetic Age With a Muscle‐Specific Epigenetic Clock

#### 2.2.1 Preprocessing of the DNA‐Methylation Datasets

Differences in preprocessing approaches can introduce variability in DNA‐methylation measurements across studies (Voisin et al. 2021). To reduce this source of bias, all datasets were downloaded and preprocessed using the same pipeline. As GSE49908 and GSE114763 data showed batch effects, batch correction with ComBat (Leek et al. 2012) was applied to these datasets. Datasets were not preprocessed jointly because age distributions varied widely (Table 1). Normalizing all datasets together could over‐ or under‐correct the DNA‐methylation measures, potentially introducing artificial variability (Voisin et al. 2021). Therefore, each dataset was preprocessed separately, and results were combined. Different versions of DNA‐methylation chips had been used across the different datasets. For the EPICv2, EPIC, and 450K datasets, raw intensities were normalized using stratified quantile normalization in the minfi package (v1.46.0) (Aryee et al. 2014) in R to correct for systematic differences between Infinium I and Infinium II probe types. HM27 datasets did not require a normalization step because this array includes only Infinium I probes with uniform signal distributions. Probes with detection *p*‐values > 0.01 in more than 50% of samples (indicating poor detection of signal above noise) and probes overlapping known single nucleotide polymorphisms were excluded based on Illumina annotation data.

**TABLE 1:** Characteristics of the former ICU patients.

Characteristic	All former ICU patients (*n* = 118)	Matched ICU patients (*n* = 97)^a^
Demographics at 5‐year follow‐up		
Age (years), median (IQR)	58 (50–66)	60 (54–68)
Male sex, *n* (%)	94 (79.6)	80 (82.4)
BMI (kg/m^2^), median (IQR)	27.3 (23.8–30.4)	27.4 (24–30.4)
White race, *n* (%)	118 (100.0)	97 (100.0)
Baseline factors at ICU‐admission		
History of diabetes, *n* (%)	10 (8.5)	9 (9.2)
History of malignancy, *n* (%)	14 (11.9)	13 (13.4)
Pre‐ICU‐admission dialysis, *n* (%)	0 (0.0)	0 (0.0)
APACHE‐II score first 24 h, median (IQR)	26 (15–32)	25 (15–32)
Admission diagnosis, *n* (%)		
Cardiac surgery	43 (36.4)	36 (37.1)
Elective other surgery	11 (9.3)	10 (10.3)
Emergency other surgery	58 (49.2)	46 (47.4)
Medical diagnosis	6 (5.1)	5 (5.2)
Sepsis upon ICU‐admission	28 (23.7)	22 (22.7)
Muscle strength at 5‐year follow‐up^b^		
HGS dominant hand (% pred), median (IQR)	93 (80–107)	96 (83–110)
HGS nondominant hand (% pred), median (IQR)	104 (89–116)	106 (91–119)
Strength shoulder (%pred), median (IQR)	93 (81–107)	91 (79–107)
Strength elbow (%pred), median (IQR)	85 (75–100)	85 (74–100)
Strength wrist (%pred), median (IQR)	98 (87–115)	98 (87–115)
Strength hip (%pred), median (IQR)	141 (122–158)	137 (126–156)
Strength knee (%pred), median (IQR)	53 (45–63)	54.0 (45–64)
Strength ankle (%pred), median (IQR)	71 (61–86)	72 (62–87)

*Note:* The matched control group had a median age of 61 years (IQR 53–70), ranged from 24 to 89 years, and was 86.5% male.

Abbreviations: %pred, percentage of predicted; APACHE‐II, acute physiology and chronic health evaluation‐II; BMI, body mass index; HGS, handgrip‐strength; ICU, intensive care unit; IQR, interquartile range.

^a^ Former ICU patients retained after propensity score matching of patients and controls for age and sex.

^b^ *p* values adjusted for age, sex, and BMI in multivariable linear regression analysis.

#### 2.2.2 Epigenetic MEAT Clock

The MEAT clock was used from the open‐access R package MEATv2 available on Bioconductor (Voisin et al. 2021). MEATv2 estimates chronological age of the general population based on DNA‐methylation status at 156 CpG sites. Using this clock, we calculated the clock‐estimated epigenetic age (DNA‐methylation age, DNAmAge) and derived the difference between DNAmAge and chronological age (DNAmAg—chronological age = “AAdiff”). DNAm age acceleration (“AAresid”) was calculated as the residuals from a linear regression of DNAmAge on chronological age using the model lm(DNAmAge~Chronological Age). This removes the linear contribution of chronological age, yielding an age‐independent measure that reflects deviation of DNAmAge from the value that is expected for a given chronological age. Positive values of AAdiff or AAresid indicate an epigenome that is older than the chronological age, whereas negative values indicate a younger epigenome. We correlated DNAmAge with chronological age with the use of Pearson correlation.

### 2.3 Impact of Epigenetic Aging as Determined With a Muscle‐Specific Epigenetic Clock on Long‐Term Altered RNA Expression Related to Long‐Term Reduced Muscle Strength After Critical Illness

We previously performed a transcriptome analysis on RNA extracted from the muscle biopsies collected during the EPaNIC 5‐year follow‐up study, retaining high‐quality data for 115 former ICU patients and 30 controls (Uzun Ayar et al. 2025). In that study, we identified 350 differentially expressed RNAs (DERNAs) in ICU patients 5 years after ICU admission as compared with controls, adjusting for age, sex and BMI (FDR < 0.05). Among these, the expression of 163 RNAs showed a significant association with the former ICU patients' long‐term reduced muscle strength.

We now assessed whether epigenetic age acceleration may (in part) statistically explain the differential expression of these 163 strength‐associated RNAs in all individuals with matched transcriptome and epigenetic age data, including 113 former ICU patients and 30 EPaNIC follow‐up controls (Figure 1). We therefore built stepwise linear regression models with the limma package (v3.62.2) in R (Ritchie et al. 2015) for each of these 163 RNAs. In step 1, we investigated whether epigenetic aging (DNAmAge, AAdiff or AAresid) independently associated with the RNA expression levels (*p* < 0.05), adjusting for age, sex and BMI. In step 2, we continued with those RNAs for which we found an association with epigenetic aging. We determined the effect sizes (β‐estimates) of having been critically ill years before on the RNA expression levels, before and after adding the epigenetic aging measure (DNAmAge, AAdiff, or AAresid) to the multivariable models and calculated the corresponding degree of change in these effect sizes.

### 2.4 Associations Between Epigenetic Age Acceleration as Determined With a Muscle‐Specific Epigenetic Clock and Muscle Strength of Former ICU Patients

Associations between epigenetic age acceleration (AAresid) and long‐term muscle strength measures as percentage of predicted for age and sex (dominant and nondominant hand, elbow, hip, shoulder, wrist, ankle, and knee) of the total cohort of former ICU patients at 5‐year follow‐up (*n* = 118) were first assessed with Pearson correlation. Next, independent associations of AAresid with long‐term muscle strength were assessed with multivariable linear regression models implemented in the limma package (Ritchie et al. 2015), adjusting for age, sex, and BMI. Associations with *p* < 0.05 were considered statistically significant.

### 2.5 Overlap of the CpG Sites and Corresponding Genes Harboring the CpG Sites of the Muscle‐Specific Clock With Long‐Term Altered DNA‐Methylation and RNA Expression After Critical Illness

We previously documented differential methylation at 7379 CpG sites in the DNA from muscle of former ICU patients 5 years after critical illness as compared with controls (Uzun Ayar et al. 2026). Overlap of those 7379 CpG sites with the 156 CpG sites used in the MEATv2 clock (Voisin et al. 2021) was determined.

The 156 CpG sites used in the MEATv2 clock (Voisin et al. 2021) map to 210 unique RNAs. We determined the overlap of those 210 RNAs with the 350 RNAs that we showed to be differentially expressed in muscle of former ICU patients 5 years after critical illness as compared with controls, and in particular the 163 RNAs that associated with impaired muscle strength of these patients (Uzun Ayar et al. 2025).

### 2.6 Statistical Analyses

Participants' characteristics, measures of muscle strength for patients, and measures of epigenetic age(ing) are presented as median and interquartile range (IQR) or number and percentage. Former ICU patients and controls were compared with the Wilcoxon signed‐rank test for continuous variables and chi‐squared test for categorical variables. Statistical significance was set at *p* < 0.05. All analyses were performed in R (v4.4.2).

## 3 Results

### 3.1 Participant Characteristics

Propensity score‐matching retained 97 former ICU patients and 97 controls. Distribution of these participants over the used datasets and corresponding age and sex distributions per dataset are shown in Table 1. The resulting patient and control cohorts were comparable for age and sex (range 24–89 years, 84.5% male, Table S2). Demographics at 5‐year follow‐up, baseline factors at ICU admission, and muscle strength measures of the total cohort of former ICU patients with DNA‐methylation data at 5‐year follow‐up, as well as of the 97‐propensity score matched former ICU patients, are shown in Table 1. These characteristics were comparable for both cohorts.

### 3.2 Epigenetic Age Estimating Chronological Age

**FIGURE 2:**
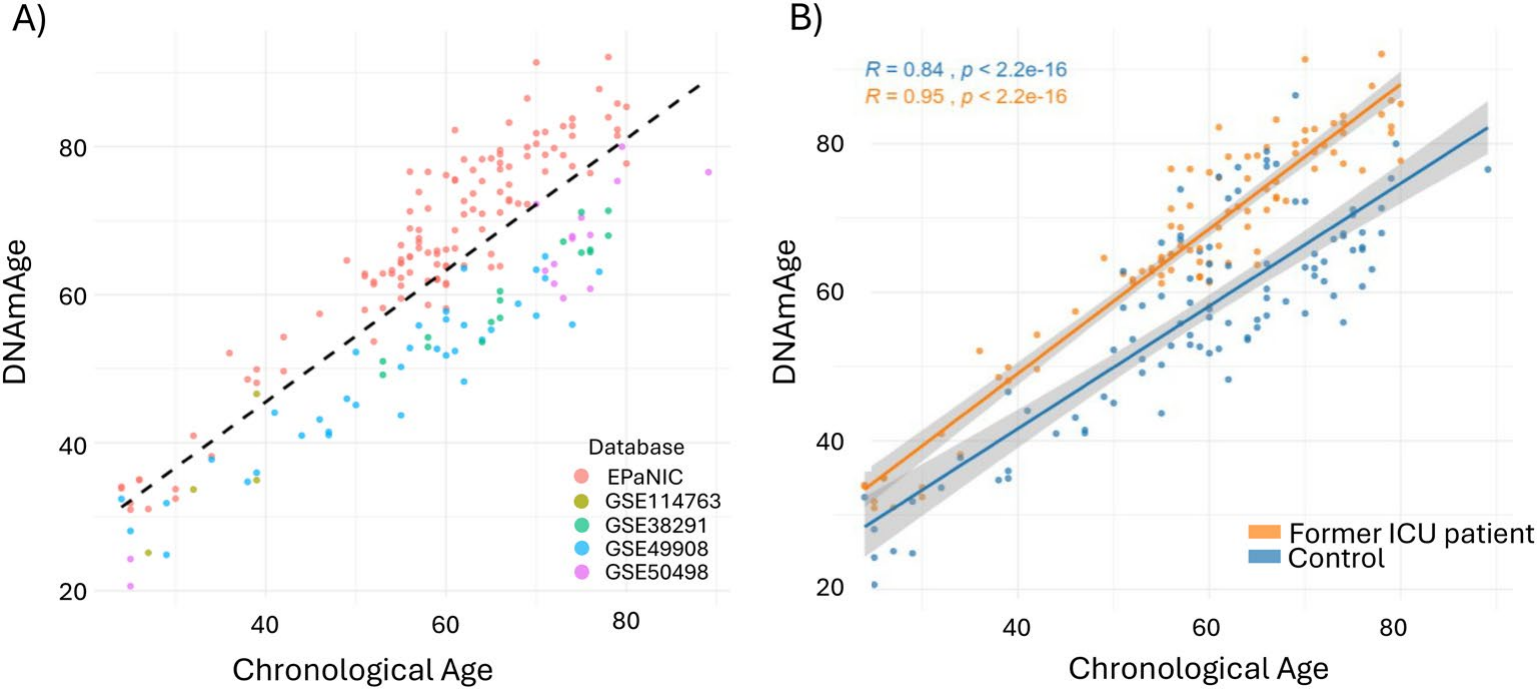
Predicted DNAmAge versus chronological age. Each point represents one of the 194 samples in the propensity score matched cohorts of former ICU patients and controls. (A) Points are colored according to the dataset of origin. The dashed black line indicates the average linear relationship. (B) Points are colored by patient (orange) versus control (blue) status. The lines represent the regression lines of the correlation between DNAmAge and chronological age for the former ICU patients and controls separately, with Pearson correlation coefficients and corresponding *p*‐values shown in the upper left side. Gray areas around the regression lines show the corresponding 95% confidence intervals.

DNAmAge estimated with the MEATv2 clock showed a high correlation with chronological age, with *R* = 0.95 for 97 patients and *R* = 0.84 for 97 controls (both *p* < 2.2 × 10^−16^; Figure 2).

### 3.3 Epigenetic Aging of Former ICU Patients as Compared With Controls

**FIGURE 3:**
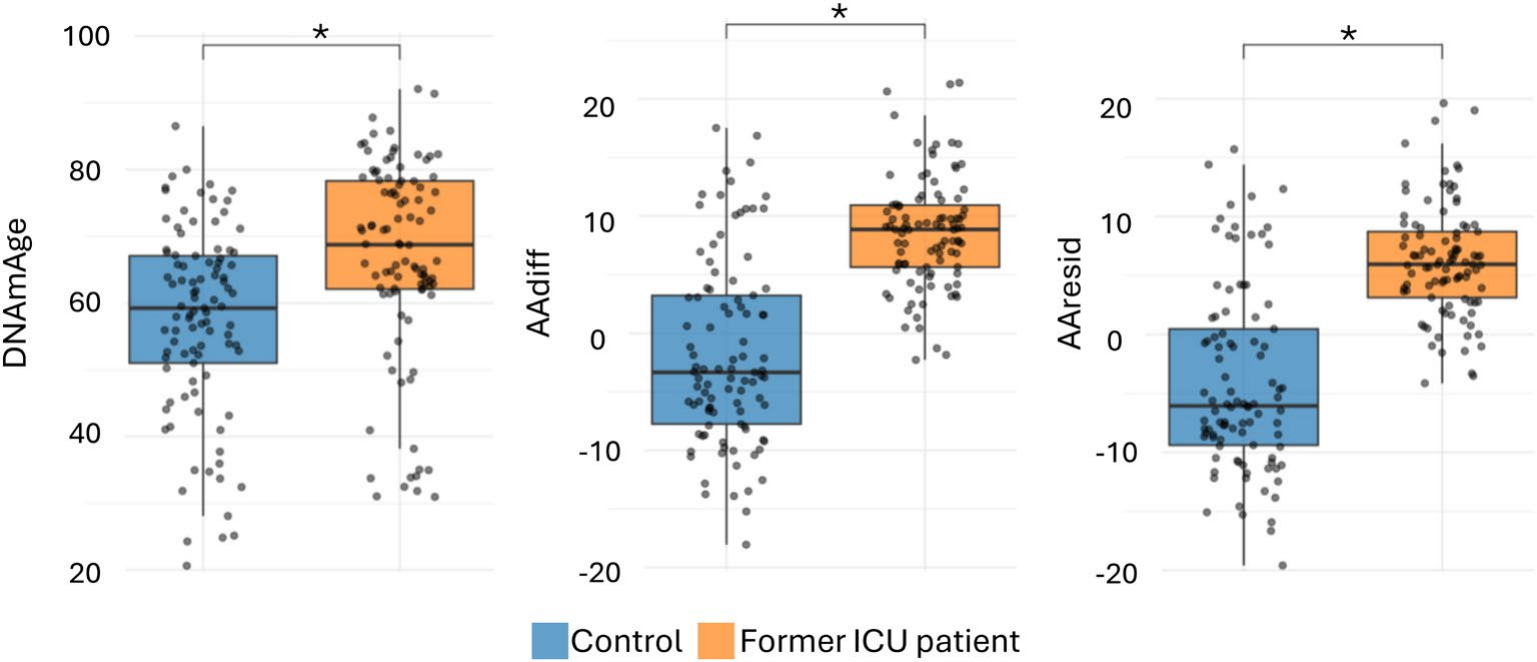
Comparison of DNAmAge, AAdiff, and AAresid in former ICU patients and controls. Epigenetic aging values are compared between former ICU patients (orange) and controls (blue). * indicates statistical significance at *p* < 2.2e‐16.

Absolute DNAmAge was significantly higher in former ICU patients as compared with age‐ and sex‐matched controls, indicating that the former ICU patients were epigenetically older than the matched controls (Figure 3). Also, the difference between the epigenetic and chronological age (AAdiff) and the age acceleration (AAresid) was larger in the former ICU patients than in the matched controls (Figure 3).

### 3.4 Epigenetic Aging as Contributor to Altered Long‐Term RNA Expression Related to Reduced Muscle Strength After Critical Illness

Epigenetic aging measures (absolute DNAmAge, AAdiff, and AAresid) independently associated with the expression level of 22 RNAs, adjusting for age, sex, and BMI, as shown in models 1 of Table S3. Effect sizes of having been critically ill years before on these 22 RNAs, adjusting for age, sex, and BMI, are shown in models 2 of Table S4. When epigenetic aging was added to these models 2, having been critically ill years before remained independently associated with the RNA expression level of these 22 RNAs, and its effect size only changed with an absolute 3.2%–8.0%.

### 3.5 Associations Between Epigenetic Age Acceleration and Muscle Strength

Raw correlations between epigenetic age acceleration (AAresid) and muscle strength of dominant hand, nondominant hand, elbow, hip, shoulder, wrist, ankle, and knee were generally weak and statistically insignificant (Table 2). Multivariable analyses adjusting for chronological age, sex, and BMI also did not reveal any significant independent associations of AAresid with the muscle strength measures (Table 2). These results indicate that epigenetic age acceleration, as measured by AAresid in muscle, was not associated with the poor long‐term muscle strength of the former ICU patients.

**TABLE 2:** Correlation and multivariable analyses adjusted for chronological age, sex, and BMI assessing the associations of AAresid with muscle strength measures.

Muscle strength	Correlation analyses		Multivariable analyses		
	R	*p*	Beta	95% CI	*P*adj^a^
Non‐dominant hand	0.021	0.825	−0.037	[−0.899, 0.825]	0.934
Dominant hand	−0.039	0.674	−0.207	[−1.015, 0.601]	0.616
Elbow	0.015	0.875	0.089	[−0.589, 0.767]	0.797
Hip	−0.142	0.130	−1.036	[−2.175, 0.103]	0.077
Ankle	0.182	0.050	0.56	[−0.077, 1.197]	0.088
Shoulder	−0.019	0.840	−0.09	[−0.872, 0.692]	0.822
Wrist	−0.07	0.451	−0.358	[−1.152, 0.436]	0.379
Knee	0.053	0.575	0.04	[−0.442, 0.522]	0.871

Abbreviation: CI, confidence interval.

^a^ *p* adjusted for chronological age, sex, and BMI.

### 3.6 Scrutiny of the MEATv2 Clock and Performance of Other Epigenetic Metrics

Since the first‐generation MEATv2, developed to predict chronological age, did not capture the functional outcome of the former ICU patients, we performed several additional analyses to shed light on this finding.

First, we took a closer look at the MEATv2 clock's composite CpG sites in relation to the previously documented alterations in DNA‐methylation and RNA expression in the former ICU patients. The clock's 156 CpG sites (Voisin et al. 2021) and the 7379 CpG sites showing altered methylation in the former ICU patients (Uzun Ayar et al. 2026) only shared six CpG sites (Table S5). Similarly, only three MEATv2 clock CpG site–associated genes were in common with the 350 differentially expressed RNAs (FDR < 0.05) in the former ICU patients (Table S6), of which only one associated with muscle strength (Uzun Ayar et al. 2025). When the threshold for differential expression was relaxed to nominal significance (*p* < 0.05), another 24 transcripts emerged, but these were not associated with muscle strength (Table S6).

Next, we additionally investigated other epigenetic metrics developed to capture systemic biological aging, including PhenoAge AAresid, principal component (PC) PhenoAge AAresid, epiTOC2 mitotic age, and Shannon entropy (Method S1). Consistent with our primary analysis, former ICU patients exhibited significantly accelerated epigenetic aging as compared with controls across all these metrics (all *p* < 0.05, Figure S1). These metrics also did not show biologically meaningful associations with reduced muscle strength measures (Table S7).

## 4 Discussion

In this study, we examined skeletal muscle epigenetic age of former ICU patients and matched controls with the muscle‐specific MEATv2 clock to determine whether former ICU patients show accelerated skeletal muscle epigenetic aging, potentially contributing to long‐term muscle weakness. Overall, a strong linear correlation was observed between chronological age and epigenetic age. Years after admission to the ICU, former ICU patients showed a significantly older epigenetic age and a clear epigenetic age acceleration as compared with controls. However, the older epigenetic age or epigenetic age acceleration did not substantially contribute to the previously documented long‐term differences in RNA expression among former ICU patients and controls. Most importantly, no associations were found between the epigenetic age acceleration in muscle of former ICU patients and the measures of their poor long‐term muscle strength.

In the first part of our hypothesis, we investigated whether skeletal muscle of former ICU patients is “epigenetically aged”, as determined by the muscle‐specific epigenetic clock MEATv2. After applying the MEATv2 clock, we first demonstrated a strong correlation between the MEATv2‐predicted DNA‐methylation age and chronological age of both former patients and controls. This confirmed consistency with previous validations of the MEATv2 clock (Jankowski et al. 2024; Sillanpaa et al. 2021). Next, we documented that, as compared with controls, former ICU patients indeed showed an older skeletal muscle epigenetic age and an epigenetic age acceleration, hence suggesting that critical illness may induce a long‐term molecular aging signature in skeletal muscle. This is in line with other studies, including those of COVID‐19 patients and sepsis patients during the ICU phase, that have documented an acute disease‐associated epigenetic age acceleration in blood cells using various epigenetic clocks (Cao et al. 2022; Sharma‐Oates et al. 2024).

Phenotypically, years after ICU discharge, former ICU patients suffer from persistent impairments in physical function, reduced exercise capacity, and poor muscle strength (Hermans et al. 2019; Van Aerde et al. 2020). We have previously associated the long‐term reduced muscle strength in this vulnerable patient population (Uzun Ayar et al. 2025) with long‐term abnormalities in the muscle transcriptome, which suggested an epigenetic molecular underlying mechanism by the documentation of genome‐wide abnormalities in DNA‐methylation (Uzun Ayar et al. 2026). The transcriptome abnormalities and further molecular and histological analyses pointed to declines in mitochondrial bioenergetics, disturbed lipid metabolism, increased collagen deposition, and muscle atrophy as most affected pathways, adjusting for age, sex, and BMI. Interestingly, these are all hallmarks of aging that have been implicated in aging‐related physical impairments (Lopez‐Otin et al. 2023). The MEATv2‐derived estimates confirmed accelerated epigenetic aging in the former ICU patients. However, the accelerated epigenetic aging did not substantially contribute to the altered transcriptome and, most importantly, did not associate with the reduced long‐term muscle strength of the former ICU patients. This suggests that the MEATv2 clock may capture global age‐related DNA‐methylation drift rather than specific, functionally relevant regulatory changes underlying long‐term muscle weakness. The lack of correlation between skeletal muscle epigenetic age acceleration and muscle strength is consistent with prior work. Indeed, none of the previous studies employing the MEAT or MEATv2 clocks observed associations between skeletal muscle epigenetic age and physiological changes, including sarcopenia (Antoun et al. 2022), physical performance (Sillanpaa et al. 2021), or exercise training (Gorski et al. 2023). The absence of such associations may reflect the original design of the MEATv2 clock, which was optimized to predict chronological rather than biological age in a general population (Voisin et al. 2021). Consequently, the CpG sites included in this muscle‐specific and many other epigenetic clocks are those most tightly correlated with chronological aging but minimally influenced by environmental or behavioral factors (Field et al. 2018). Supporting this interpretation, only six of the CpG sites used to calculate epigenetic age with the MEATv2 clock overlapped with the altered muscle DNA‐methylome of the former ICU patients for which functional relevance was suggested (Table S5) (Uzun Ayar et al. 2026). Similarly, only three MEATv2 clock CpG site–associated genes overlapped with the altered transcriptome of the former ICU patients, of which only one associated with muscle strength (Uzun Ayar et al. 2025). The minimal overlap at both the epigenetic and transcriptomic levels reinforces that the MEATv2 clock predominantly captures chronological methylation patterns rather than functionally relevant epigenetic remodeling. Collectively, these findings underscore the need for a muscle‐specific epigenetic clock that integrates biological and functional parameters, similar to the GrimAge and PhenoAge models developed for blood (Levine et al. 2018; Lu et al. 2019). We tested several epigenetic aging metrics developed for blood, including the second‐generation PhenoAge and PC PhenoAge clocks, the epiTOC2 mitotic clock, and Shannon entropy. They all confirmed significantly accelerated biological aging in former ICU patients but failed to show biologically meaningful correlations with muscle strength. This again stresses that new muscle‐specific approaches, trained on health‐related and performance‐based outcomes, are needed to more accurately reflect the biological dimension of muscle aging, particularly in conditions associated with long‐term muscle weakness.

The strengths of our study include the long time window of follow‐up after critical illness, the use of a validated, muscle‐specific epigenetic clock (Voisin et al. 2021) and combination with complementary second‐generation and mitotic aging metrics, as well as the integration of DNA‐methylation data from post‐ICU patients with transcriptomic profiles and detailed functional assessments that allowed multi‐omics and biological interpretation. In addition, the inclusion of additional control samples from public datasets improved the robustness of the comparison. Importantly, patient and control groups were well matched for chronological age and sex. Also, several limitations should be acknowledged. First, critical illness is largely unpredictable, precluding collection of muscle biopsies prior to ICU admission. Thus, even though patients who clearly showed impaired physical function already before ICU admission were excluded from this study, and alterations in DNA‐methylation can arise de novo during critical illness (Guiza et al. 2020), it remains possible that part of the accelerated epigenetic aging in these ICU survivors already occurred before ICU admission. Second, information on physical function obtained at the time of the 5‐year follow‐up is consistent with reduced physical activity and a more sedentary lifestyle in the former ICU patients, with worse scores on the SF‐36 physical functioning domain which captures the extent to which health limits physical activities and a shorter 6‐min walk distance as a measure of functional capacity (Hermans et al. 2019). It remains unclear whether critical illness‐related molecular changes limit physical capacity, thereby leading to a more sedentary lifestyle, or whether sedentary behavior drives DNA‐methylation changes. Third, not all public healthy subject samples included comprehensive baseline characteristics beyond age and sex, introducing possible confounding. However, a sensitivity analysis with additional adjustment for known history of diabetes or malignancy (considered to be absent if not known) yielded similar results (data not shown). Finally, the overall sample size, although large for this type of study, remained modest. Therefore, we cannot exclude that lack of significant association of epigenetic aging with muscle strength may relate to type II error. However, the previously observed associations of transcriptomic alterations, in part explained by DNA‐methylation changes, with muscle strength in the same patient cohort, as well as lack of association of skeletal muscle epigenetic age with physiological changes in other studies (Antoun et al. 2022; Sillanpaa et al. 2021), Gorski et al. (2023) may argue against this. The invasive nature of muscle biopsies in general continues to limit large‐scale cohort studies and the further refinement of muscle‐specific epigenetic clocks.

In conclusion, years after critical illness, former ICU patients showed accelerated skeletal muscle epigenetic aging. However, the current muscle‐specific epigenetic clock MEATv2 appeared to capture chronological rather than functional aging and did not substantially contribute to the long‐term altered RNA expression nor the long‐term muscle weakness. Future research should aim to develop biologically informed, outcome‐based muscle‐specific clocks integrating multi‐omics and clinical outcomes to elucidate the molecular mechanisms underlying long‐term muscle weakness in ICU survivors and to identify potential therapeutic targets to mitigate long‐term muscle weakness.

## Author Contributions

C.U.A., G.V.B., and I.V. designed the study and corresponding data analyses. C.U.A., I.D., and I.V. gathered the data. C.U.A. and I.V. statistically analyzed the data. C.U.A., G.V.B., and I.V. wrote the manuscript. All authors read and approved the final manuscript.

## Funding

This work was supported by the Research Foundation—Flanders [Fonds Wetenschappelijk Onderzoek (FWO)], Belgium (research grant to I.V. (G039912), and research grant to I.V. and G.V.B. (G017325N)), by the Methusalem program of the Flemish government (through the KU Leuven to G.V.B. and I.V., METH14/06) and by European Research Council Advanced Grants (AdvG‐2012‐321670, AdvG‐2017‐785809 and AdvG‐2023‐101133276) to G.V.B. Part of the study was funded by the European Union. Views and opinions expressed are however those of the author(s) only and do not necessarily reflect those of the European Union or the European Research Council. Neither the European Union nor the granting authority can be held responsible for them.

## Ethics Statement

The Leuven University Hospital Ethics Committee approved the study protocol and informed consent forms (ML4190). The study has thus been performed in accordance with the ethical standards laid down in the 1964 Declaration of Helsinki and its later amendments. All participants gave their informed consent prior to inclusion in the study.

## Conflicts of Interest

The authors declare no conflicts of interest.

## Supporting information


**Table S1:** Detailed information about the datasets used in this study.
**Table S2:** Characteristics of the cohorts used in this study.
**Table S3:** Independent associations of epigenetic age measures with gene expression outcomes (adjusted for age, sex, and BMI).
**Table S4:** Effect estimates for former patients versus controls before (Model 2) and after (Model 3) adjustment for epigenetic age measures.
**Table S5:** Six CpG sites shared between previously identified DMPs and the 156 CpGs used by MEATV2 for epigenetic age prediction.
**Table S6:** Genes shared between the 210 genes harboring CpGs used by MEATV2 for epigenetic age prediction and those differentially expressed in former ICU patients vs. controls.
**Table S7:** Correlation analysis between epigenetic aging metrics and muscle strength measures.

**Figure S1:** Accelerated biological aging across multiple epigenetic metrics.
**Methods S1**. Additional epigenetic aging metrics.

## Data Availability

The GSE114763, GSE38291, GSE49908, and GSE50498 are available in the Gene Expression Omnibus repository (https://www.ncbi.nlm.nih.gov/geo). The individual‐level data from EPaNIC cannot be publicly shared due to ethical approval. Data sets and other documents may be shared on reasonable request under the format of future collaborative projects that further elaborate on this specific research topic. Proposals for collaborative projects must be directed to the corresponding author and will be considered for approval. The data set required to address the research question will then only be shared after signing of a data access agreement. Data are located in controlled access data storage at KU Leuven.
