# Supplementary material for: Epigenetic Aging of Critical Illness Survivors Assessed by the Muscle‐Specific “Clock” and Its Relationship With Reduced Long‐Term Muscle Strength
Source: Aging Cell. 2026 Jul 12;25(7):e70629. doi: 10.1111/acel.70629 (PMC13356824; doi:10.1111/acel.70629)
Supplement: Supplementary file 2 — Figure S1: Accelerated biological aging across multiple epigenetic metrics. Methods S1. Additional epigenetic aging metrics. [file ACEL-25-e70629-s002.docx]

**SUPPLEMENTARY APPENDIX**

**Epigenetic aging of critical illness survivors assessed by the muscle-specific “clock” and its relationship with reduced long-term muscle strength**

Ceren **Uzun Ayar**, M.Sc.^1^, Inge **Derese**, B.Sc.^1^,

Greet **Van den Berghe**, M.D.^1,2,#^, Ilse **Vanhorebeek**, Ph.D.^1,#^

^1^ Laboratory of Intensive Care Medicine, Department of Cellular and Molecular Medicine, KU Leuven, 3000 Leuven, Belgium; ^2^ Clinical Division of Intensive Care Medicine, University Hospitals Leuven, 3000 Leuven, Belgium; ^#^ Equally contributed

Running title: Epigenetic muscle aging of ICU survivors

**Corresponding author**: Ilse Vanhorebeek, MEng, PhD Laboratory of Intensive Care Medicine, KU Leuven, Herestraat 49, B-3000 Leuven, Belgium; Tel +32 16 330532; Fax +32 16 344015; email [ilse.vanhorebeek@kuleuven.be](mailto:ilse.vanhorebeek@kuleuven.be).

**Supplementary Methods………………………………………………………………...……3**

**Methods S1**. Additional epigenetic aging metrics...……………………….……………...…..4

**Supplementary Figures…………………………………………..………………….……..…5**

**Figure S1.** Accelerated biological aging across multiple epigenetic metrics….…............……6

**References**………………………………………………...……………………………...……7

**Supplementary Methods**

**Method S1. Additional epigenetic aging metrics**

We calculated additional epigenetic metrics to capture systemic biological aging. The second-generation PhenoAge clock and its noise-reduced version, principal component (PC) PhenoAge are trained to reflect multi-system physiological decline and mortality risk rather than chronological age alone (Higgins-Chen et al., 2022; Levine et al., 2018), based on the methylation status of 513 CpG sites. epiTOC2 is a mitotic clock that estimates mitotic age as the cumulative number of stem cell divisions based on DNA methylation changes at 163 Polycomb group target CpG sites (Teschendorff, 2020). Finally, Shannon entropy is used as a measure of epigenetic disorder across the methylome, making use of all available CpG sites (Xie et al., 2011). All metrics were computed from normalized DNA methylation data using the methylCIPHER R package. PhenoAge, PC PhenoAge, epiTOC2, and Shannon entropy were calculated using the corresponding functions implemented in this package. Age acceleration measures (AAresid) were calculated for PhenoAge and PC PhenoAge as residuals from linear regression models of epigenetic age on chronological age. As these clocks were primarily developed in blood, they are interpreted here as measures of systemic rather than muscle specific biological aging. Associations of epigenetic age acceleration (AAresid), mitotic age and Shannon entropy with long term muscle strength, expressed as percentage of predicted values adjusted for age and sex for dominant and nondominant hand, elbow, hip, shoulder, wrist, ankle, and knee, in the total cohort of former ICU patients at 5 year follow up (n = 118) were assessed with Pearson correlation.

**Supplementary Figures**


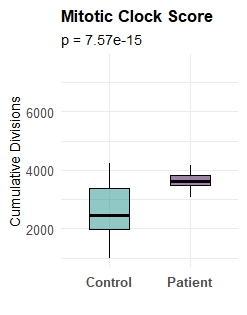

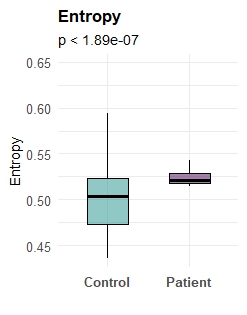

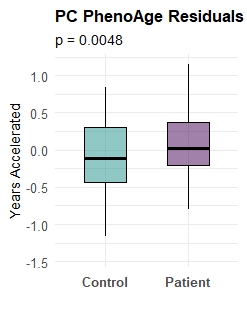

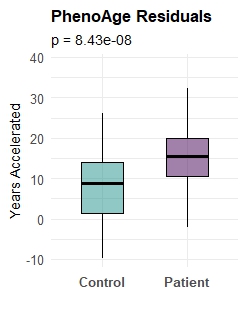


A.

C.

D.

B.

**Figure S1.** **Accelerated biological aging across multiple epigenetic metrics.** Comparison of four distinct epigenetic aging measures between controls (blue) and former ICU patients (purple): **A.** PhenoAge residuals (second-generation clock) capturing systemic physiological decline; and **B.** PC PhenoAge residuals (noise-reduced version of PhenoAge). **C.** epiTOC2 (mitotic clock) representing cumulative stem cell divisions; **D.** Shannon entropy as a measure of epigenetic disorder/noise. All metrics consistently demonstrate significantly accelerated biological aging in former ICU patients as compared with controls (P < 0.05). Data are presented as boxplots, with boxes showing median and interquartile range and whiskers representing the minimum and maximum values.

**References**

1. Higgins-Chen, A. T., Thrush, K. L., Wang, Y., et al. 2022. “A computational solution for bolstering reliability of epigenetic clocks: Implications for clinical trials and longitudinal tracking.” Nat Aging 2, no. 7: 644-661. <https://doi.org/10.1038/s43587-022-00248-2>
2. Levine, M. E., Lu, A. T., Quach, A., et al. 2018. “An epigenetic biomarker of aging for lifespan and healthspan.” Aging-Us 10, no. 4: 573-591. <https://doi.org/10.18632/aging.101414>
3. Teschendorff, A. E. 2020. “A comparison of epigenetic mitotic-like clocks for cancer risk prediction.” Genome Med 12, no. 1: 56. <https://doi.org/10.1186/s13073-020-00752-3>
4. Xie, H., Wang, M., de Andrade, A., et al. 2011. “Genome-wide quantitative assessment of variation in DNA methylation patterns.” Nucleic Acids Research 39, no. 10: 4099-4108. <https://doi.org/10.1093/nar/gkr017>
